# Social Mobility, Health and Wellbeing in Poland

**DOI:** 10.3389/fsoc.2021.736249

**Published:** 2021-11-25

**Authors:** Olga Zelinska, Alexi Gugushvili, Grzegorz Bulczak

**Affiliations:** ^1^ Institute of Philosophy and Sociology, Polish Academy of Sciences, Warsaw, Poland; ^2^ Department of Sociology and Human Geography, University of Oslo, Oslo, Norway

**Keywords:** social mobility, self-reported health, psychological wellbeing, Poland, diagonal reference models

## Abstract

Recently there has been a surge of interest in the consequences of intergenerational social mobility on individuals’ health and wellbeing outcomes. However, studies on the effects of social mobility on health, using high-quality panel survey data, have almost exclusively been conducted in Western welfare democracies. To account for this gap, and using empirical data from one of the largest and most eventful post-communist countries, Poland, in this study we investigate how individuals’ origin and destination socio-economic position and social mobility are linked to self-rated health and reported psychological wellbeing. We use the Polish Panel Survey (POLPAN) data to construct self-rated health and psychological wellbeing measures, origin, destination and occupational class mobility variables, and account for an extensive set of sociodemographic determinants of health. We employ diagonal reference models to distinguish social mobility effects from origin and destination effects, and account for possible health selection mechanisms. Our results suggest that there is an occupational class gradient in health in Poland and that both parental and own occupational class matter for individual health outcomes. We also find a positive reported psychological wellbeing effect for upward social mobility from the working to the professional class.

## Introduction

Recently there has been a surge of interest in the consequences of intergenerational social mobility on individuals’ health and wellbeing outcomes (Simandan, 2018; Kaiser and Trinh, 2021). The attention on the topic is driven by the increasing availability of high-quality data, ongoing methodological debate on how to detect mobility effects, and corresponding advances in statistical software (van der Waal et al., 2017; Kaiser, 2018; Präg, 2019). Studies on the consequences of social mobility on health have been almost exclusively conducted in Western welfare democracies (Cardano et al., 2004; Iveson and Deary, 2017), yet the major changes in terms of industrialization and fundamental economic restructuring have happened in post-communist societies in Central and Eastern Europe. These countries first experienced a decline in their overall economic output in the early 1990s, followed by often a rapid recovery (King et al., 2009). This is especially true for nations that joined the European Union in 2004. Such transitional economies have also experienced major replacement of the “free” health care systems by predominantly market-oriented health care provision and have been characterized by mass anxiety connected to the overall politico-economic transformations. In this study, using empirical data from one of the largest and most eventful post-communist countries, Poland, we investigate how individuals’ origin and destination socio-economic position and social mobility experiences are linked to their health and psychological wellbeing outcomes.

Poland has been referred to as a “laboratory of structuring processes” (Janicka and Słomczyński, 2014). Following the collapse of the Communist system, the country faced major changes in the nature of its economy, the role of government, and the structure of employment (Kolodko, 2009). Two contrasting hypotheses were proposed to explain the implications of these fundamental changes for social mobility patterns in post-communist societies (Bukodi and Goldthorpe, 2010). On the one hand, a departure from the affirmative actions for disadvantaged groups, over-employment of the labor force, and forceful equalization of rewards during communist times would increase intergenerational reproduction of advantages and disadvantages in the post-communist period (Jackson and Evans, 2017; Gugushvili, 2017). On the other hand, the country’s transit from a command economy to a market economy, accompanied by the restoration of democratic institutions, could provide greater opportunities for many to experience social mobility in a more meritocratic society (Domański, 2005; Gugushvili, 2015).

Empirical testing of these two theoretical perspectives has revealed a complex picture. The communist societies, including Poland, during the late communist period, were not substantively different from the capitalist countries in terms of their modes of intergenerational social mobility (Gugushvili, 2017). The market economy, which is arguably associated with more meritocratic principles of distribution of rewards, did not diminish the role of individuals’ parental characteristics in their life chances. In Poland, the pace of changes has also been non-linear and has differed in different periods following systemic changes (Domański, 2004a). Moreover, differentiated adjustment to the post-communist transition has created both “winners” and “losers” from the transformation, i.e., those who ended up at the top and the bottom of various wellbeing indicators (Słomczyński et al., 2007).

For both privileged and disadvantaged, social and economic transformation has had a profound but very different impact on individual attitudes, orientations, and lifestyles. Those who have ended up at the top of the social hierarchy tend to support democracy and the market system. They feel they have realized their plans and believe that poverty is a personal rather than a structural problem. Simultaneously, those who have been less successful are also nostalgic for socialism, do not feel accomplished, and support greater redistribution of resources (Słomczyński et al., 2007; Gugushvili, 2016; Gugushvili et al., 2020).

An enormous amount of stress has been the main wellbeing threat in the transformation process. The psychosocial aspect of wellbeing has been linked with material and socio-political aspects of change (Leinsalu et al., 2009). An illustrative study has connected respondents’ anxiety to increasing social and income disparities, and to the major contrast between the financial difficulties of the majority of Poles and the abnormally high earnings of the fewer transitional “winners” (Watson, 2004). In short, economic and social uncertainties have contributed to lower wellbeing in Poland (Brzezinski, 2019).

### Social Determinants of Health and Wellbeing in Poland

In addition to changing objective material conditions and changes in many subjective aspects of life, post-communist changes have, as expected, contributed to deteriorating health and growing inequalities in the Polish society (Raphael, 2006). According to a 1995 survey, 85% of Poles believed that the state of health in the country had decreased substantially, and a third of respondents assessed their own health as bad or very bad (Ostrowska, 1999). The country experienced an increase in psychological problems, including rising suicide rates and alcoholism (Watson, 2004). The economic recession at the end of the 1990s had caused the poorest social groups to limit their utilization of expensive specialized medical services despite their evident need (Golinowska and Sowa, 2006).

As material conditions improved, especially after Poland joined the European Union in 2004, the country has experienced steady growth in life expectancy and an improved general state of health of the population (World Health Organization, 2012). Yet, these improvements have further increased inequalities in health for various social groups.

Studies have revealed substantial gender, socio-economic, and regional inequalities in health in Poland (Szaflarski, 2001; World Health Organization, 2012; Tobiasz-Adamczyk and Zawisza, 2017). Although there is no consensus on biological and socio-cultural explanations of health inequality between men and women (Frąckowiak-Sochańska, 2010; Królikowska, 2011), there are significant differences between gender-specific social mobility patterns. More recent research has identified major differences in health status between those with different education levels (Korzeniowska and Puchalski, 2015). Acceptance of healthier lifestyles has led to better health among the more educated. Simultaneously, the number of those who report bad or very bad health has increased among individuals with lower education (Sowa, 2011). In Wrocław, the fourth largest city in Poland, mortality rates were the highest and the lowest among persons with, respectively, primary and tertiary education. This gradient was more pronounced among males than females (Brajczewski and Rogucka, 1993). For adult females, educational attainment had a strong independent association with their body mass index (Szklarska and Jankowska, 2003).

Material wellbeing is another crucial determinant of inequalities in health. Social mobility is often an indicator of the attainment of economic resources that shape access to health-conducive lifestyles and living conditions (Ostrowska, 2011; World Health Organization, 2012). Among elderly Poles, both physical and psychological aspects of health are strongly related to individuals’ socio-economic position, measured by income and self-assessed social position (Mikucka, 2016). Another factor impacting health inequalities relates to social ties. In addition to the positive effects of social connections with friends or religious institutions (Ostrowska, 2011), marital status is a significant predictor of health in Poland (Kludacz-Alessandri and Cygańska, 2020).

Poland is one of the least urbanized countries in Europe, and substantial differences in health have been shown by settlement type and geographic location (Ucieklak-Jeż and Bem, 2020). Polish men in cities live longer than men in rural areas (Sowa, 2011). Nonetheless, a World Health Organization (WHO) report suggests that urban or rural place of residence has relatively little impact, while the region of residence is of greater significance for differences in mortality (World Health Organization, 2012). Another study examines the differences in health among the residents of both urban and rural areas across six NUTS (Nomenclature of Units for Territorial Statistics) regions in Poland (Tobiasz-Adamczyk and Zawisza, 2017). It concludes that the differences may be attributed both to the urban-rural split and to the region of residence.

### Impact of Social Mobility on Health and Wellbeing

Although a growing number of studies look at how social mobility in different parts of the world affects health and wellbeing, very few of those are conducted in post-communist countries, including Poland (Gugushvili et al., 2019b; Gugushvili and Präg, 2020; Präg and Gugushvili, 2020). Limited evidence exists about the effect of social origin on health, such as a study showing that the share of respondents who reported bad health in the capital, Warsaw, was almost double among those whose parents had primary education only compared with those with highly educated parents (Ostrowska, 2011). Another study has found that intergenerational educational mobility modified coronary heart disease risk in middle-aged Polish men but not in women. Beneficial effects were particularly strong in those men who improved their educational attainment compared with their fathers (Jankowska et al., 2008). Further, a comparative study of self-assessed health in post-communist European countries, including Poland, has demonstrated the benefits of upward mobility and the detrimental impact of downward mobility on health (Campos-Matos and Kawachi, 2015).

In addition to statistical challenges in identifying independent effects of social mobility on health and wellbeing, the described studies are also limited to investigating mobility in educational attainment. Scholars specializing in the Polish context, however, point out the benefits of using occupational class to determine an individual’s position within the social structure in this country (Janicka, 2020). Following a sociological tradition of studying social stratification and mobility patterns through individuals’ labor market situation and their employment relations (Blau and Duncan, 1967; Erikson and Goldthorpe, 1992), this study aims to analyze what the health implications of social origin and destination positions are, and social mobility between these two positions in terms of the occupational class to which individuals belong.

### Hypotheses

Occupational class of both individuals and their parents can have an independent health effect because the occupational group to which an individual belongs indicates how stable and secure her or his employment and corresponding income is (or was), and how much income that individual’s parents received during their childhood (Goldthorpe et al., 1980). In other words, social class reflects how well individuals can satisfy their own needs, which are essential for explaining health outcomes (Marmot, 2005). Although there have been major upgrades in occupational class structure from parental to offspring generations in post-communist Poland, we expect that both origin and destination social classes are significantly associated with individuals’ health.

Our expectations related to social class mobility are in line with previous research on this topic. Whenever studies find a significant association between different types of downward social mobility and health, it is almost exclusively negative (Nicklett and Burgard, 2009; Na-Ek and Demakakos, 2017; Euteneuer and Schäfer, 2018). The “falling from grace” thesis (Newman, 1988) might be suitable for describing why downward social mobility during post-communist transition leads to adverse health implications. On the other hand, the post-communist transition process was also associated with upward social mobility experiences in a large share of the population (Bukodi et al., 2020). The major social, cultural and psychological stress involved in upward social mobility would lead to various health concerns, as predicted by the dissociative thesis (Sorokin, 1927). Alternatively, there are reasons to believe that upward social mobility can be beneficial for health if it promotes locus of control, self-confidence, and a sense of achievement and gratitude, as predicted by the “from rags to riches” thesis (Gugushvili et al., 2019c).

## Materials and Methods

### Dataset

We use the Polish Panel Survey, POLPAN, one of the longest continuously run panel studies in Central and Eastern Europe that focuses on individuals’ life course, socio-economic structure, and inequality (Tomescu-Dubrow et al., 2021). The longitudinal design of the survey means POLPAN’s data are well suited for research on the health consequences of intergenerational social mobility (Zelinska et al., 2021). Initiated in 1987-88, POLPAN has been fielded in 5-year intervals, most recently in 2018 (Wave VII). The core of the sample consists of panel respondents. Since 1998, the sample of panelists has been complemented with randomly drawn renewal samples of young adults. In this way, waves maintain a representative age distribution for the country at the time of any given POLPAN survey. Response rates for full panelists are consistently above 70%, but vary for intermittent panelists and the young.

In the present study, we use POLPAN waves I (1988) through VI (2013). The choice of Wave VI over the more recent Wave VII (2018) is due to a greater number of respondents. We measured the health- and wellbeing-related outcome variables and the majority of controls as of 2013, using only those respondents who participated in the 2013 Wave. The information on respondents’ social origin, father’s occupation when a respondent was 14 years old, has been asked several times since 1988. While its value should ideally stay the same across the different measuring points, respondents often provided slightly different assessments. Consequently, to construct the variable of parental occupational class, we used the earliest instance of reporting.

Wave VI was fielded in 2013 with the total number of completed interviews being N = 2,780 through pen and paper personal interviews using a standardized questionnaire (with a mailed questionnaire being used when face-to-face interview was not possible). These data contain 2,670 valid observations for self-rated health and 2,531 observations for psychological wellbeing. Controlling for parental occupational attainment reduced the sample size to 2,063 observations for health and 1,927 for wellbeing outcomes. Further accounting for own occupational attainment and other control variables, we obtained working samples of 1,853 and 1,891 observations for self-assessed health and psychological wellbeing, respectively, after listwise deletion of observations with missing information. Table with household income, presented in Supplementary Material, have a further reduced sample size.

### Dependent Variables

**FIGURE 1:**
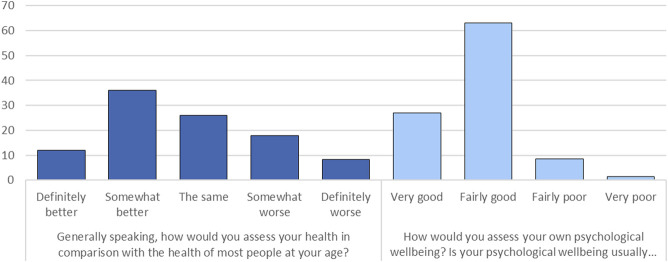
Self-reported health and psychological wellbeing in the analyzed sample, %.

For dependent variables, we identified two measures of self-assessed health and psychological wellbeing. The 2013 POLPAN questionnaire contains the following two items. The first, “Generally speaking, how would you assess your health in comparison with the health of most people at your age?” has five answer options ranging from “definitely better” to “definitely worse.” This question is different from a more conventional self-rated health question as it contains an element of comparison to others, and should consequently be interpreted accordingly. Earlier research suggests that social comparison to family members, friends, neighbors, and other more distant groups (e.g., those learned about in the media) is an important implication for individuals’ health and wellbeing (Gugushvili et al., 2019a; Gugushvili and Reeves, 2021). The second question asks: “How would you assess your own psychological wellbeing? Is your psychological wellbeing usually … ”, with four answers ranging from “very good” to “very poor.” To ease interpretation and understanding of results, the original scales of both of the dependent variables were reversed and renamed. The self-assessed health ranges from “very bad” (=1) to “very good” (=5), and psychological wellbeing—from “very bad” (=1) to “very good” (=4). Figure 1 presents the distribution of the outcome variables and suggests that most people positively assess their health and psychological wellbeing.

### Social Origin, Destination, and Mobility

We used the information about POLPAN participants’ and their fathers’ occupations to construct social origin, destination, and mobility variables. We rely only on the data on fathers’ occupation because POLPAN has not collected data on mothers’ occupation consistently across waves. In particular, we considered information about which of the 14 socio-occupational groups the fathers belonged to when respondents were 14 years old. One of the main reasons for selecting this particular categorization was its availability for both respondents and their fathers. Moreover, this classification is rooted in the historical and cultural context of the communist and post-communist Polish society (Domański et al., 2009). It was designed as a standard analytical tool for social stratification researchers in Poland (Domański, 2004b).

For fathers, we have used information from the earliest instance of reporting (data comes from Waves I through VI). Following the earlier work (Domański et al., 2009), we then recoded the initial 14-category variables into three hierarchical classes: 1) salariat class, which includes professionals, high-level officials and managers; 2) intermediate class, which includes technical specialists, administrative workers and middle-level specialists, business owners and technicians; and 3) working class, which includes workers and farmers. Individuals’ destination class was constructed from their occupational category at POLPAN wave VI (2013). We looked at respondents’ main job and took a similar approach as we did for fathers’ occupations by generating three hierarchical occupational categories.

We operationalize social mobility as a difference between origin and destination occupational categories. The distribution of individuals’ and their fathers’ occupational categories as well as all nine possible trajectories of intergenerational mobility are visualized in Table 1.

**TABLE 1:** Intergenerational mobility trajectories in the analyses sample.

Fathers’ class	Respondents’ class			
	Salariat	Intermediate	Working	Total
Salariat	↔ 1.3%	↓ 1.7%	↓↓ 1.4%	4.4%, N = 94
	Immobile	One-step downward	Two-steps downward	
Intermediate	↑2.3%	↔7.4%	↓ 6.5%	16.6%, N = 342
	One-step upward	Immobile	One-step downward	
Working	↑↑ 3.7%	↑ 19.3%	↔ 56.3%	79.4%,N = 1,680
	Two-steps upward	One-step upward	Immobile	
Total	7.3%, N = 154	28.4%, N = 602	64.3%, N = 1,360	100.0%

Comparing the occupational structure between parental and offspring generations suggests that Poland experienced almost a doubling within the professional occupations, a significant increase in intermediate occupations, and a 15.1% decrease in the share of the working class. Still, the latter group of occupations remains by far the biggest class category. Table 1 also indicates that most individuals are immobile (65.0%), and that many experienced upward mobility (25.3%), while some also moved down in the social hierarchy (9.6%). For the multivariable analysis, we take a nuanced approach by creating, respectively, two-steps downward, one-step downward, one-step upward, and two-steps upward mobility variables. For example, a person coming from a farmer’s family and occupying a managerial position in 2013 would be two-steps upwardly mobile, while those who came from a professional’s family and ended up as middle-level specialists would be classified as being one-step downwardly mobile.

### Control Variables

In line with the previous research on socio-economic determinants of health in Poland, all estimated models account for the age of individuals (both continuous and squared terms for curvilinearity) and gender (male = 1). We also include a set of control variables for respondents’ marital status (married = 1), residential area (urban = 1), and region (six Polish regions according to NUTS classification). Additionally, we consider respondents’ educational attainment in 2013 (1 = primary; 2 = secondary and vocational; 3 = tertiary). We also provide the analysis with household income in 2013 (standardized) as an additional control variable of health in the Supplementary Material.

Studies on social mobility indicate that accounting for individuals’ initial health status is important due to possible health selection effects. POLPAN does not include information on individuals’ childhood health, but all POLPAN respondents in 2013 were asked about their height. Adult’s body height can be considered as a crude indicator of individuals’ health and wellbeing during childhood, as it is known that the most important non-genetic factors affecting adult body height are early life nutrition and diseases (Meyer and Selmer, 1999; Silventoinen, 2003; Chen and Li, 2009). Therefore, we account for individuals’ height as a crude proxy for their initial health. Based on the absolute measure of height, we generated quartiles calculated separately by gender and 10-years birth cohorts. Next, we created binary variables for those whose height fell within the bottom quarter and for those whose height appeared in the top quarter of the distribution in the respective gender and birth cohort (Jarosz and Gugushvili, 2020). The described relative height indicator of those who are the shortest or tallest among their peers is a crude measure of individuals’ initial health. We present descriptive statistics for all variables in Supplementary Table S1 in the supplementary materials.

### Statistical Analysis

To understand the effect of intergenerational occupational mobility on individuals’ health and psychological wellbeing, we employed diagonal reference models (DRM) (Sobel, 1981). Widely used in social mobility research (Houle and Martin, 2011; Billingsley et al., 2018; Gugushvili et al., 2021), DRMs allow disentangling of the effects of mobility on the impact of origin and destination positions. DRM first estimates intercepts for immobile individuals whose social origin position equals their destination position. These intercepts then are used by DRM to derive a weight parameter (p) representing the importance of the origin (parental) social position relative to that of the destination (own) position, which takes values between 0 and 1. Values greater than 0.5 indicate that origin position matters more than destination (individual’s) position in determining individual health and wellbeing outcomes. On the other hand, if this weight parameter equals 0 the position of origin has no effect, while the position of destination does. DRM’s coefficients are interpreted in the same manner as conventional regression coefficients.

Our outcome variables for self-reported health and psychological wellbeing are ordinal, but existing software solutions that fit DRMs do not allow the running of regression specifications which would be equivalent to ordered or generalized ordered logistic regressions (Williams, 2006). One option would be to dichotomize our outcome variables and fit DRMs with the logit link function. Nonetheless, a recent Monte Carlo simulation of the DRM approach suggests that the non-linear specification of this method produces greater bias than linear specification. This bias is especially severe for weight parameters, and upward mobility coefficients are under-estimated, while estimates with linear specifications are within acceptable boundaries (Procopio and Samuel, 2020). Hence, we fit DRMs with the linear link function.

Our models demonstrate the effects of respondents’ own and parental class position and social mobility on self-reported health and psychological wellbeing outcomes. In the baseline model, we control only for age and gender. In the following models, we introduce marital status, urban locality and the region of residence. We then introduce social mobility parameters, add a proxy variable for initial health, and account for educational attainment. When DRMs include dummy variables for both upward and downward social mobility, their reference category are individuals who do not experience social mobility. The Supplementary Material provide the analysis with income added as an additional control variable and interaction terms between social mobility parameters and age, gender, and education. All estimates were performed using the “drm” module in Stata 16 software (Kaiser, 2018). Because of the nature of our data and the fact that the outcome variables have different scales, the sizes of the coefficients for self-rated health and psychological wellbeing cannot be directly compared. Thus, in what follows, we will concentrate on the signs and significance of the coefficients across the different models rather than on their specific values.

## Results

### Is There an Occupational Class Gradient in Health and Wellbeing Among Immobile Groups?

We start our analysis by describing the occupational class gradient in health and wellbeing outcomes. We do this by comparing the health of intergenerationally immobile individuals in professional, intermediate and working-class occupations. The results presented in Tables 2, 3 suggest that an occupational class gradient in health among immobile individuals is present in Poland. The difference is much more pronounced between working-class and other classes than between intermediate and professional classes for both health and psychological wellbeing outcomes. The negative effect of being intergenerationally immobile in the working class is somewhat reduced when a set of sociodemographic controls (Model 2), the proxy variable for initial health (Model 4), and individuals’ educational attainment are accounted for (Model 5).

**TABLE 2:** Base and full models for self-rated health.

	Model 1	Model 2	Model 3	Model 4	Model 5
Intercept	4.27^***^ (0.23)	4.16^***^ (0.24)	4.16^***^ (0.25)	4.10^***^ (0.25)	4.06^***^ (0.26)
Intergenerationally immobile					
Professional class	0.19 (0.10)	0.14 (0.10)	0.10 (0.12)	0.09 (0.12)	0.08 (0.13)
Intermediate class	0.14 (0.07)	0.12 (0.07)	0.17^*^ (0.09)	0.17^*^ (0.09)	0.14 (0.09)
Working class	−0.32^***^ (0.06)	−0.27^***^ (0.06)	−0.27^***^ (0.07)	−0.26^***^ (0.07)	−0.22^**^ (0.08)
Weight parameters					
Origin	0.55^***^ (0.11)	0.60^***^ (0.13)	0.72^**^ (0.28)	0.70^*^ (0.28)	0.84^*^ (0.39)
Destination	0.45^***^ (0.11)	0.40^**^ (0.13)	0.28 (0.28)	0.30 (0.28)	0.16 (0.39)
Sociodemographic controls					
Age in 2013	−0.03^**^ (0.01)	−0.03^**^ (0.01)	−0.03^**^ (0.01)	−0.03^**^ (0.01)	−0.03^**^ (0.01)
Age^2^	0.00^**^ (0.00)	0.00^**^ (0.00)	0.00^**^ (0.00)	0.00^**^ (0.00)	0.00^**^ (0.00)
Gender; 2 = female	−0.13^*^ (0.05)	−0.11^*^ (0.05)	−0.11^*^ (0.05)	−0.12^*^ (0.05)	−0.13^*^ (0.05)
Married	––––	0.09 (0.06)	0.08 (0.06)	0.08 (0.06)	0.07 (0.06)
Urban	––––	0.18^***^ (0.06)	0.18^**^ (0.06)	0.18^**^ (0.06)	0.16^**^ (0.06)
NUTS region					
Central	––––	reference			
South	––––	0.01 (0.08)	0.01 (0.08)	0.01 (0.08)	0.02 (0.08)
East	––––	−0.02 (0.08)	−0.02 (0.08)	−0.02 (0.08)	−0.02 (0.08)
North-West	––––	−0.05 (0.08)	−0.05 (0.08)	−0.05 (0.08)	−0.03 (0.08)
South-West	––––	0.12 (0.11)	0.12 (0.11)	0.12 (0.11)	0.13 (0.11)
North	––––	0.04 (0.09)	0.04 (0.09)	0.03 (0.09)	0.05 (0.09)
Mobility dummies					
Downward two-step mobility	––––	––––	−0.02 (0.28)	−0.04 (0.28)	−0.08 (0.31)
Downward one-step mobility	––––	––––	−0.11 (0.14)	−0.11 (0.14)	−0.13 (0.15)
Upward one-step mobility	––––	––––	−0.00 (0.13)	−0.01 (0.13)	−0.01 (0.14)
Upward two-step mobility	––––	––––	0.18 (0.17)	0.17 (0.17)	0.16 (0.19)
Height					
Short	––––	––––	––––	0.08 (0.06)	0.08 (0.06)
Tall	––––	––––	––––	0.21^**^ (0.07)	0.21^**^ (0.07)
Education					
Primary	––––	––––	––––	––––	−0.17^*^ (0.07)
Secondary and vocational	––––	––––	––––	––––	reference
Tertiary	––––	––––	––––	––––	−0.03 (0.08)
AIC	5676.99	5674.79	5680.90	5675.15	5673.08
Number of observations	1853	1853	1853	1853	1853

Notes: ^*^ *p* < 0.05, ^**^ *p* < 0.01, ^***^ *p* < 0.001, standard errors in parentheses.

**TABLE 3:** Base and full models for reported psychological wellbeing.

	Model 1	Model 2	Model 3	Model 4	Model 5
Intercept	4.11^***^ (0.12)	4.18^***^ (0.13)	4.09^***^ (0.13)	4.07^***^ (0.13)	3.99^***^ (0.14)
Intergenerationally immobile					
Professional class	0.10^*^ (0.05)	0.09 (0.05)	−0.00 (0.06)	−0.00 (0.06)	−0.05 (0.07)
Intermediate class	0.05 (0.04)	0.05 (0.04)	0.09^*^ (0.05)	0.09^*^ (0.05)	0.08 (0.05)
Working class	−0.15^***^ (0.03)	−0.14^***^ (0.03)	−0.09^*^ (0.04)	−0.09^*^ (0.04)	−0.04 (0.04)
Weight parameters					
Origin	0.41^**^ (0.14)	0.45^**^ (0.15)	0.86^**^ (0.31)	0.85^**^ (0.31)	0.94^*^ (0.38)
Destination	0.59^***^ (0.14)	0.55^***^ (0.15)	0.14 (0.31)	0.15 (0.31)	0.06 (0.38)
Sociodemographic controls					
Age in 2013	−0.02^***^ (0.00)	−0.03^***^ (0.01)	−0.02^***^ (0.01)	−0.02^***^ (0.01)	−0.02^***^ (0.01)
Age^2^	0.00^**^ (0.00)	0.00^***^ (0.00)	0.00^***^ (0.00)	0.00^***^ (0.00)	0.00^**^ (0.00)
Gender; 2 = female	−0.09^**^ (0.03)	−0.08^**^ (0.03)	−0.08^**^ (0.03)	−0.08^**^ (0.03)	−0.09^**^ (0.03)
Married	––––	0.07^*^ (0.03)	0.07^*^ (0.03)	0.07^*^ (0.03)	0.06 (0.03)
Urban	––––	0.01 (0.03)	0.01 (0.03)	0.01 (0.03)	−0.00 (0.03)
NUTS region					
Central	––––	reference			
South	––––	−0.05 (0.04)	−0.05 (0.04)	−0.05 (0.04)	−0.05 (0.04)
East	––––	−0.04 (0.04)	−0.04 (0.04)	−0.04 (0.04)	−0.04 (0.04)
North-West	––––	−0.13^**^ (0.05)	−0.14^**^ (0.04)	−0.14^**^ (0.04)	−0.13^**^ (0.05)
South-West	––––	0.04 (0.06)	0.04 (0.06)	0.04 (0.06)	0.04 (0.06)
North	––––	−0.02 (0.05)	−0.03 (0.05)	−0.03 (0.05)	−0.02 (0.05)
Mobility dummies					
Downward two-step mobility	––––	––––	0.10 (0.15)	0.09 (0.15)	0.13 (0.15)
Downward one-step mobility	––––	––––	0.03 (0.07)	0.03 (0.07)	0.05 (0.07)
Upward one-step mobility	––––	––––	0.09 (0.06)	0.09 (0.06)	0.06 (0.05)
Upward two-step mobility	––––	––––	0.25^**^ (0.08)	0.25^**^ (0.08)	0.17^*^ (0.08)
Height					
Short	––––	––––	––––	0.04 (0.03)	0.04 (0.03)
Tall	––––	––––	––––	0.05 (0.04)	0.05 (0.04)
Education					
Primary	––––	––––	––––	––––	−0.08^*^ (0.04)
Secondary and vocational	––––	––––	––––	––––	reference
Tertiary	––––	––––	––––	––––	0.05 (0.05)
AIC	3464.33	3461.39	3460.88	3462.13	3459.13
Number of observations	1891	1891	1891	1891	1891

Notes: ^*^ *p* < 0.05, ^**^ *p* < 0.01, ^***^ *p* < 0.001, standard errors in parentheses.

### How Important are the Origin and Destination Classes?

We now examine the relative importance of individuals’ origin and destination social class in explaining their self-rated health and psychological wellbeing outcomes. The results in Tables 2, 3 suggest that parental social class consistently plays an important role in individuals’ health outcomes regardless of their social mobility characteristics. In fact, in all models for self-rated health, parental occupation matters more than individuals’ occupational class. In turn, for reported psychological wellbeing (Models 1 and 2), we observe that the weight for individuals’ own social class is higher than the weight for the parental class. However, this also changes when individuals’ social mobility parameters (Model 3) and educational attainment (Model 5) are accounted for. DRM estimates for psychological wellbeing suggest that most of the variation in the outcome variables is explained by fathers’ occupational class. This indicates that social mobility should be appropriately accounted for to accurately estimate the relative role of own and parental characteristics in determining health outcomes.

### Are There Social Mobility Effects on Health and Wellbeing?

After describing occupational class gradient in health and relative importance of origin and destination social class, DRM models also allow us to test if there are net social mobility effects on health independent of origin and destination class positions. Models 3-5 account for four social mobility parameters: one-step and two-steps downward and upward mobility experiences and their estimated coefficients are compared with immobile individuals in our analytical sample. We find that three out of four mobility parameters are not significantly linked to self-rated health and reported psychological wellbeing outcomes. However, we observe a consistent effect for two-steps upward social class mobility on psychological wellbeing. This effect holds after all controls are accounted for, including individuals’ educational attainment, and the size of this effect (0.17, *p* < 0.05) is greater than the effects identified for gender, educational attainment, or regional belonging. The improvement in the model fit with social mobility parameters is also demonstrated by the lower values of the Akaike Information Criterion (AIC) in Models 3 and 5. The significant effect of two-steps upward social class mobility also remains after accounting for household income (Supplementary Table S2), yet income itself is not a significant explanation of self-reported health or psychological wellbeing in Poland.

### Do Control Variables Have Different Effects on Self-Rated Health and Psychological Wellbeing?

Our analysis also reveals a nuanced picture of how control variables play a different role in explaining self-rated health and psychological wellbeing outcomes. As expected, age and gender are important for health. Older respondents report worse health, but at a certain point this association reverses. Men report worse health compared with women. Marital status is linked to better self-assessed health, but this association is only significant for reported psychological wellbeing when educational attainment is not accounted for. Residing in the Polish north-western region, compared with residence in the country’s central region, is associated with worse psychological wellbeing. Simultaneously, city dwellers report better health compared with rural residents. The individuals’ height variable, which we use as a proxy for initial health, is significantly associated with physical health. This is also shown by a significant reduction of AIC value in Model 4, Table 2. Being in the top 25% of the height distribution for a specific gender and birth cohort is associated with better health, and the effect of this association is 0.21 (*p* < 0.01). The results also indicate that those with only primary education evaluate their own health and psychological wellbeing as worse than those with secondary education.

### Do Mobility Effects Vary by Sociodemographic Groups?

We estimated DRMs with the interaction terms between mobility parameters and a set of covariates of health and wellbeing: gender, age, and educational attainment. We did not find any statistically significant interaction terms between mobility trajectories and age or educational attainment. Simultaneously, interaction terms of two-steps downward mobility for females are significant at *p* < 0.05, for both health and psychological wellbeing (0.89 and 0.47, respectively). Supplementary Tables S3, S4 in the supplementary materials include the details. This suggests that a two-steps downward intergenerational social class mobility has a different effect on the self-assessed health and psychological wellbeing of females than it does for males. Apparently, women in Poland suffer less from the negative health consequences connected to steep downward social mobility.

### Limitations of the Study

We recognize the multiple limitations of this study. Since POLPAN waves are also designed as stand-alone cross-sectional surveys, in response to various research design considerations, each wave uses multiple questionnaire versions. Thus, different groups of POLPAN respondents have answered different versions of the questionaries, limiting the number of observations for further statistical analysis. This problem is especially acute in the case of “sensitive” questions (e.g., related to personal income), which by default suffer from limited response rates in surveys. Additionally, the structure of employment during the communist regime, which included a large working-class population but also a very small professional class, produced a highly uneven distribution for respondent’s origin class. For example, in our working sample, those stemming from the professional background are only 4.4%, or 94 individuals. We also recognize potential concerns with statistical power in our analysis related to small numbers, and that there is a need to conduct analysis with larger samples in future research. Additionally, there was an unfortunate change in the scale for self-rated health from four to five items and, as a result, the estimates for the two outcome variables cannot be directly compared. Last but not least, father’s occupation data is available in POLPAN across waves, while information on mothers’ characteristics is extremely limited in this dataset.

## Discussion

This study has aimed to contribute to the existing scholarship on the consequences of intergenerational social mobility on individuals’ health and psychological wellbeing outcomes. One of the primary motivations for the analysis was the relative lack of studies on social mobility effects on health and wellbeing that had used high-quality panel data, employed recent statistical developments, and focused on post-communist societies.

The results from the DRM approach suggest that there is a social class gradient in health in Poland. For both health and psychological wellbeing outcomes, the difference is much more pronounced between working-class and other occupations than between intermediate and professional classes. Those who come from working-class families and have a working-class occupation report worse health and psychological wellbeing. Next, childhood conditions, formed largely according to father’s socio-occupational position within the Polish socialist society, are more important for self-rated health and psychological wellbeing in adult life than an individual’s own place within the occupational hierarchy. For reported psychological wellbeing, we observe that the weight of own occupational attainment is higher than for parental attainment, but this relationship reverses when individuals’ social mobility parameters and educational attainment are accounted for.

We also observe systemic and consistent effects for upward social class mobility for psychological wellbeing. Stemming from a working-class background and attaining a position in the professional class is associated with better psychological wellbeing. The relationship between occupational mobility and self-assessed health differs by gender. Our estimations suggest that women suffer less from the negative health consequences connected to downward social mobility. Coming from a family with a father belonging to the professional class, but occupying a working-class position is associated with better self-reported health and psychological wellbeing in women compared with men who experienced the same mobility trajectory.

The results of our study should be interpreted in the light of the changes that Poland experienced in the 1990s and after 2004. The 1990s transformation resulted in major changes in the country’s social structure, previously characterized by a large portion of the population working in state agricultural farms or in “hidden unemployment” (Balcerowicz, 1994). Long-lasting unemployment (Balcerowicz, 2005) and massive rural to urban migration (Kaczmarczyk and Okólski, 2008) contributed to high levels of uncertainty with a noticeable adverse impact on social capital (Dzialek, 2009). Social networks and family ties were often disrupted, and trust levels declined (Lehman and Sztompka, 2001). This period has put pressure on individuals’ health and particularly on their psychological wellbeing (Leka and Nicholson, 2019).

At the same time, social changes also produced fertile ground for social upgrading to occur. Many had to adjust to the new situation by acquiring new skills facilitated by booming private and state-run higher education or by starting their own businesses (Balcerowicz, 2008). Entrepreneurship flourished in the 1990s giving many the experience of self-control and success (Hunter, 2018).

Arguably, breaking the established social ties, apart from immediate lack of social support, was also likely to be associated with measurable benefits, as our analysis shows. Upwardly mobile individuals may have been exposed to different, likely more healthy lifestyles. Why we do not see a significant effect of the short-range mobility on health is an open question. It seems plausible that, for this group of individuals, the benefits and costs cancel out. Probably, in this context, only long-range upward mobility brings benefits large enough to compensate for the associated costs. Because we observe mobility effects only in psychological wellbeing, it is reasonable to speculate that this effect can be largely explained through an increased sense of self-control, self-satisfaction, and happiness (Tumin, 1957; Watkins et al., 2003).

Our results show that, after mobility is accounted for, origin characteristics play a more important role than the destination. Parental influences have a long-lasting effect on individuals’ health. Norms and ties associated with a parental class are more important in determining individuals’ health and psychological wellbeing outcomes than their own professional attainment. This was expected in the light of both past research and theoretical explanations (Steiber, 2019). We observe the respondents in a time of great uncertainty, with parents being a likely source of financial and non-financial support (Cox et al., 1997). Poland’s social structure changed rapidly during the analyzed period, during which we observe persistent differences in the subjective health assessment among the lowest social classes. This finding fits well with previous research showing that the subjective assessment of the old system is noticeably different between the privileged and the disadvantaged—transformation “winners” and “losers” (Słomczyński et al., 2007). It can be argued that the lowest social class’s pessimistic view about the transformation is also connected to lower ratings for self-assessed health, which may reflect both actual health problems and increased pessimism.

Our results on gendered health perception and social mobility are in line with the literature on the Post-Soviet region, which suggests that opportunity structures and social mobility in these countries have resulted in different experiences for men and women (Urbaeva, 2019; Azarova et al., 2017). Our findings, at the same time, should be interpreted with caution. Existing research suggests that women, compared with men, often list more life goals, including those that account for family needs (Williams et al., 2013). In pursuing these goals, women anticipate more negative outcomes connected to professional growth (e.g., life-career trade-off). As a result, they place less importance on self-interested power-related goals (Gino et al., 2015), so that suffering from a loss in terms of socio-occupational group downgrading would not be as traumatic for women as it is for men. This explanation, however, is far from satisfying. We do not know how these respondents self-assessed their advancement or downgrading in the social hierarchy. Studies show that subjective mobility assessment performs better in explaining individuals’ characteristics than do objective indicators (Präg and Gugushvili, 2021). We also do not know what the reference point for such assessment would be—is it father’s or mother’s job? Higher education, a usual attribute of professional class family background, might have mitigated the effects of occupational downward mobility on health for women. While we have only limited information on mothers’ occupation available in POLPAN, a further exploration into how contextual opportunity structures have influenced women’s views about their wellbeing, mobility, and their roles in family and society in general is necessary.

## Data Availability

Publicly available dataset, POLPAN, was analyzed in this study. The dataset can be downloaded from the following link: https://dataverse.harvard.edu/dataset.xhtml?persistentId=doi:10.7910/DVN/DAPH0P.

## References

[B89] Azarova A. Darja I. Alexi G. Mihaly F. Scheiring G. Horvat p. (2017). The Effect of Rapid Privatisation on Mortality in Mono-Industrial Towns in Post-Soviet Russia: A Retrospective Cohort Study. The Lancet Public Health 2(5), e231–38. 10.1016/S2468-2667(17)30072-5 28626827 PMC5459934

[B1] Balcerowicz L. (2008). How Capitalism Was Built. The Transformation of Central and Eastern Europe, Russia, and Central Asia. Anders Åslund. Eurasian Geogr. Econ. 49, 228–230. 10.2747/1539-7216.49.2.228

[B2] Balcerowicz L. (2005). Post-communist Transition: Some Lessons. SSRN J. 10.2139/ssrn.676661

[B3] Balcerowicz L. (1994). Understanding Postcommunist Transitions. J. Democracy 5, 75–89. 10.1353/jod.1994.0053

[B4] Billingsley S. Drefahl S. Ghilagaber G. (2018). An Application of diagonal Reference Models and Time-Varying Covariates in Social Mobility Research on Mortality and Fertility. Soc. Sci. Res. 75, 73–82. 10.1016/j.ssresearch.2018.06.008 30080493

[B5] Blau P. M. Duncan O. D. (1967). The American Occupational Structure. New York: Wiley and Sons.

[B6] Brajczewski C. Rogucka E. (1993). Social Class Differences in Rates of Premature Mortality Among Adults in the City of Wrocław, Poland. Am. J. Hum. Biol. 5, 461–471. 10.1002/ajhb.1310050410 28548402

[B7] Brzezinski M. (2019). Diagnosing Unhappiness Dynamics: Evidence from Poland and Russia. J. Happiness Stud. 20, 2291–2327. 10.1007/s10902-018-0044-6

[B8] Bukodi E. Goldthorpe J. H. (2010). Market versus Meritocracy: Hungary as a Critical Case. Eur. Sociological Rev. 26, 655–674. 10.1093/esr/jcp043

[B9] Bukodi E. Paskov M. Nolan B. (2020). Intergenerational Class Mobility in Europe: A New Account. Soc. Forces 98, 941–972. 10.1093/sf/soz026

[B10] Campos-Matos I. Kawachi I. (2015). Social Mobility and Health in European Countries: Does Welfare Regime Type Matter. Soc. Sci. Med. 142, 241–248. 10.1016/j.socscimed.2015.08.035 26318213

[B11] Cardano M. Costa G. Demaria M. (2004). Social Mobility and Health in the Turin Longitudinal Study. Soc. Sci. Med. 58, 1563–1574. 10.1016/S0277-9536(03)00354-X 14759699

[B12] Chen Y. Li H. (2009). Mother's Education and Child Health: Is There a Nurturing Effect. J. Health Econ. 28, 413–426. 10.1016/j.jhealeco.2008.10.005 19058866

[B13] Cox D. Jimenez E. Okrasa W. (1997). Family Safety Nets and Economic Transition: A Study of Worker Households in Poland. Rev. Income Wealth 43, 191–209. 10.1111/j.1475-4991.1997.tb00214.x

[B14] Domański H. (2004a). O Ruchliwości Społecznej W Polsce. Warszawa: Wydawnictwo IFIS PAN.

[B15] Domański H. Sawiński Z. Słomczyński K. M. (2009). Sociological Tools Measuring Occupations. New Classification and Scales. Warsaw: IFIS Publishers.

[B16] Domański H. (2004b). Struktura Spoleczna. Warszawa: Wydawnictwo Naukowe „Scholar”.

[B17] Domański H. (2005). The Polish Transformation. Eur. J. Soc. Theor. 8, 453–470. 10.1177/1368431005056423

[B18] Dzialek J. (2009). Social Capital and Economic Growth in Polish Regions. Available at: https://mpra.ub.uni-muenchen.de/18287/1/Social_capital_and_economic_growth_in_Polish_regions_JDzialek_MPRA.pdf [Accessed October 27, 2021].

[B19] Erikson R. Goldthorpe J. H. (1992). The Constant Flux: A Study of Class Mobility in Industrial Societies. Oxford; New York: Clarendon Press; Oxford University Press.

[B20] Euteneuer F. Schäfer S. J. (2018). Brief Report: Subjective Social Mobility and Depressive Symptoms in Syrian Refugees to Germany. J. Immigr Minor. Health 20, 1533–1536. 10.1007/s10903-018-0692-y 29340877

[B21] Frąckowiak-Sochańska M. (2010). Rodzinne I Społeczno-Kulturowe Uwarunkowania Zaburzeń Psychicznych. Analiza Z Perspektywy Płci Społeczno-Kulturoioej. Rocz. Socjol. Rodz. 20, 153–183.

[B22] Gino F. Wilmuth C. A. Brooks A. W. (2015). Compared to Men, Women View Professional Advancement as Equally Attainable, but Less Desirable. Proc. Natl. Acad. Sci. U S A. 112, 12354–12359. 10.1073/pnas.1502567112 26392533 PMC4603465

[B23] Goldthorpe J. H. Llewellyn C. Payne C. (1980). Social Mobility and Class Structure in Modern Britain. Oxford: Oxford University Press.

[B24] Golinowska S. Sowa A. (2006). Health and Morbidity in the Accession Countries Country Report. CEPS Available at: https://www.ceps.eu/ceps-publications/health-and-morbidity-accession-countries-country-report-poland/ [Accessed October 15, 2020].

[B25] Gugushvili A. Bulczak G. Zelinska O. Koltai J. (2021). Socioeconomic Position, Social Mobility, and Health Selection Effects on Allostatic Load in the United States. PLoS One 16, e0254414. 10.1371/journal.pone.0254414 34347798 PMC8336836

[B26] Gugushvili A. Jarosz E. McKee M. (2019a). Compared with Whom? Reference Groups in Socio-Economic Comparisons and Self-Reported Health in 34 Countries. Int. J. Epidemiol. 48, 1710–1720. 10.1093/ije/dyz122 31730706

[B27] Gugushvili A. McKee M. Murphy M. Azarova A. Irdam D. Doniec K. (2019b). Intergenerational Mobility in Relative Educational Attainment and Health-Related Behaviours. Soc. Indic. Res. 141, 413–441. 10.1007/s11205-017-1834-7 31467460 PMC6694039

[B87] Gugushvili A. (2017). Political Democracy, Economic Liberalization, and Macro-Sociological Models of Intergenerational Mobility. Social Science Research 66, 58–81. 10.1016/j.ssresearch.2017.06.003 28705364

[B28] Gugushvili A. Reeves A. (2021). How Democracy Alters Our View of Inequality - and what it Means for Our Health. Soc. Sci. Med. 283, 114190. 10.1016/j.socscimed.2021.114190 34242889

[B29] Gugushvili A. Reeves A. Jarosz E. (2020). How Do Perceived Changes in Inequality Affect Health. Health Place 62, 102276. 10.1016/j.healthplace.2019.102276 32479355

[B30] Gugushvili A. Zhao Y. Bukodi E. (2019c). 'Falling from grace' and 'rising from Rags': Intergenerational Educational Mobility and Depressive Symptoms. Soc. Sci. Med. 222, 294–304. 10.1016/j.socscimed.2018.12.027 30677643

[B31] Gugushvili A. (2017). Change or Continuity? Intergenerational Social Mobility and post-communist Transition. Res. Soc. Stratification Mobility 52, 59–71. 10.1016/j.rssm.2017.10.004

[B32] Gugushvili A. (2015). Economic Liberalization and Intergenerational Mobility in Occupational Status. Comp. Sociol. 14, 790–820. 10.1163/15691330-12341368

[B33] Gugushvili A. (2016). Intergenerational Social Mobility and Popular Explanations of Poverty: A Comparative Perspective. Soc. Just Res. 29, 402–428. 10.1007/s11211-016-0275-9

[B34] Gugushvili A. Präg P. (2021). Intergenerational Social Mobility and Health in Russia: Mind over Matter. Adv. Life Course Res. 47, 100390. 10.1016/j.alcr.2020.100390 36695147

[B35] Houle J. N. Martin M. A. (2011). Does Intergenerational Mobility Shape Psychological Distress? Sorokin Revisited. Res. Soc. Stratif. Mobil. 29, 193–203. 10.1016/j.rssm.2010.11.001 25152556 PMC4139926

[B36] Hunter, Jr. R. J. (2018). Poland's Sustained "March to a Market Economy": The Choice between Competing Visions and Plans. Rwe 9, 61–76. 10.5430/rwe.v9n1p61

[B37] Iveson M. H. Deary I. J. (2017). Intergenerational Social Mobility and Subjective Wellbeing in Later Life. Soc. Sci. Med. 188, 11–20. 10.1016/j.socscimed.2017.06.038 28692825

[B38] Jackson M. Evans G. (2017). Rebuilding Walls: Market Transition and Social Mobility in the Post-Socialist Societies of Europe. SocScience 4, 54–79. 10.15195/v4.a3

[B39] Janicka K. (2020). Samoocena Ruchliwości Międzypokoleniowej W Kontekście Przemian Struktury Społecznej. StudiaBAS 2, 129–142. 10.31268/StudiaBAS.2020.17

[B40] Janicka K. Słomczyński K. (2014). Struktura Społeczna W Polsce: Klasowy Wymiar Nierówności. Przegląd Socjol. 63 (LXIII), 55–72.

[B41] Jankowska E. A. Szklarska A. Lipowicz A. Lopuszańska M. Koziel S. Bielicki T. (2008). Inter-generation Social Mobility Modifies Framingham Risk Score in Polish Middle-Aged Men, but Not in Women. J. Biosoc. Sci. 40, 401–412. 10.1017/S0021932007002635 18093347

[B42] Jarosz E. Gugushvili A. (2020). Parental Education, Health Literacy and Children's Adult Body Height. J. Biosoc. Sci. 52, 696–718. 10.1017/s0021932019000737 31722763

[B43] Kaczmarczyk P. Okólski M. (2008). Demographic and Labour-Market Impacts of Migration on Poland. Oxford Rev. Econ. Pol. 24, 599–624. 10.1093/oxrep/grn029

[B44] Kaiser C. (2018). DRM: Stata Module to Fit Sobel’s Diagonal Reference Model (DRM). Available at: https://econpapers.repec.org/RePEc:boc:bocode:s458506 .

[B45] Kaiser C. Trinh N. A. (2021). Positional, Mobility, and Reference Effects: How Does Social Class Affect Life Satisfaction in Europe. Eur. Sociol. Rev. 37, 713–730. 10.1093/esr/jcaa067

[B46] King L. Hamm P. Stuckler D. (2009). Rapid Large-Scale Privatization and Death Rates in Ex-Communist Countries: An Analysis of Stress-Related and Health System Mechanisms. Int. J. Health Serv. 39, 461–489. 10.2190/HS.39.3.c 19771951

[B47] Kludacz-Alessandri M. Cygańska M. (2020). “Socioeconomic Determinants of Health Status Among Older Adults in Poland,” in Eurasian Economic Perspectives. Editors Bilgin M. H. Danis H. Demir E. (Cham: Springer International Publishing), 239–253. 10.1007/978-3-030-53536-0_17

[B48] Kolodko G. W. (2009). A Two-Thirds of success. Poland's post-communist Transformation 1989-2009. Communist Post-communist Stud. 42, 325–351. 10.1016/j.postcomstud.2009.07.005

[B49] Korzeniowska E. Puchalski K. (2015). “Nierówności Edukacyjne a Zachowania Zdrowotne I Zdrowie,” in Socjologia Medycyny W Polsce Z Perspektywy Półwiecza. Editors Ostrowska A. Skrzypek M. (Warszawa: Wydawnictwo Instytutu Filozofii i Socjologii PAN).

[B50] Królikowska S. (2011). Nierówności W Stanie Zdrowia Między Kobietami a Mężczyznami W Kontekście Płci Biologicznej Oraz Społeczno-Kulturowej. Acta Univ. Lodz. Folia Sociol. 39, 33–52.

[B51] Lehman E. W. Sztompka P. (2001). Trust: A Sociological Theory. Contemp. Sociol. 30, 418. 10.2307/3089802

[B52] Leinsalu M. Stirbu I. Vågerö D. Kalediene R. Kovács K. Wojtyniak B. (2009). Educational Inequalities in Mortality in Four Eastern European Countries: Divergence in Trends during the post-communist Transition from 1990 to 2000. Int. J. Epidemiol. 38, 512–525. 10.1093/ije/dyn248 19052117

[B53] Leka S. Nicholson P. J. (2019). Mental Health in the Workplace. Occup. Med. (Lond) 69, 5–6. 10.1093/occmed/kqy111 30753713

[B54] Marmot M. (2005). Social Determinants of Health Inequalities. Lancet 365, 1099–1104. Mar 19-25. 10.1016/S0140-6736(05)71146-6 15781105

[B55] Meyer H. E. Selmer R. (1999). Income, Educational Level and Body Height. Ann. Hum. Biol. 26, 219–227. 10.1080/030144699282723 10355493

[B56] Mikucka M. (2016). “The Health of Elderly Men and Women,” in Social Inequality and the Life Course (Warsaw: IFiS Publishers), 269–293. Available at: http://polpan.org/wp-content/uploads/2014/04/POLPAN_2_Social_Inequality_and_the_Life_Course.pdf .

[B57] Na-Ek N. Demakakos P. (2017). Social Mobility and Inflammatory and Metabolic Markers at Older Ages: the English Longitudinal Study of Ageing. J. Epidemiol. Community Health 71, 253–260. 10.1136/jech-2016-207394 27647138

[B58] Newman K. S. (1988). Falling from grace: The Experience of Downward Mobility in the American Middle Class. New York: Free Press.

[B59] Nicklett E. J. Burgard S. A. (2009). Downward Social Mobility and Major Depressive Episodes Among Latino and Asian-American Immigrants to the United States. Am. J. Epidemiol. 170, 793–801. 10.1093/aje/kwp192 19671834 PMC2768522

[B60] Ostrowska A. (2011). Psychospołeczne Uwarunkowania Nierówności W Zdrowiu. Zdr. Publiczne I Zarządzanie 9, 55–63. 10.4467/20842627OZ.11.017.0554

[B61] Ostrowska A. (1999). Styl Życia a Zdrowie: Z Zagadnień Promocji Zdrowia. Warszawa: Wydawnictwo IFiS PAN.

[B88] Präg P. Gugushvili A. (2020). Intergenerational Social Mobility and Self-Rated Health in Europe. SocArxiv. 10.31235/osf.io/5tk4z

[B62] Präg P. Gugushvili A. (2021). Subjective Social Mobility and Health in Germany. Eur. Soc., 1–23. 10.31235/osf.io/x3bzk

[B63] Präg P. (2019). Visualizing Individual Outcomes of Social Mobility Using Heatmaps. Socius 5, 237802311985548. 10.1177/2378023119855486

[B64] Procopio A. Samuel R. (2020). “Is it Origin, Destination or Mobility? A Monte Carlo Simulation of the Diagonal Reference Model,” in Cambridge Social Stratification Research Seminar (Cambridge).

[B65] Raphael D. (2006). Social Determinants of Health: Present Status, Unanswered Questions, and Future Directions. Int. J. Health Serv. 36, 651–677. 10.2190/3MW4-1EK3-DGRQ-2CRF 17175840

[B66] Silventoinen K. (2003). Determinants of Variation in Adult Body Height. J. Biosoc. Sci. 35, 263–285. 10.1017/S0021932003002633 12664962

[B67] Simandan D. (2018). Rethinking the Health Consequences of Social Class and Social Mobility. Soc. Sci. Med. 200, 258–261. 10.1016/j.socscimed.2017.11.037 29301638

[B68] Słomczyński K. M. Janicka K. Shabad G. Tomescu-Dubrow I. (2007). Changes in Class Structure in Poland, 1988-2003: Crystallization of the Winners - Losers’ Divide. Polish Sociol. Rev. 157, 45–64.

[B69] Sobel M. E. (1981). Diagonal Mobility Models: A Substantively Motivated Class of Designs for the Analysis of Mobility Effects. Am. Sociological Rev. 46, 893. 10.2307/2095086

[B70] Sorokin P. A. (1927). Social Mobility. New York: Harper & Brothers.

[B71] Sowa A. (2011). Społeczne Uwarunkowania Stanu Zdrowia W Polsce. Zdr. Publiczne I Zarządzanie 9, 28–37.

[B72] Steiber N. (2019). Intergenerational Educational Mobility and Health Satisfaction across the Life Course: Does the Long Arm of Childhood Conditions Only Become Visible Later in Life. Soc. Sci. Med. 242, 112603. 10.1016/j.socscimed.2019.112603 31655463

[B73] Szaflarski M. (2001). Gender, Self-Reported Health, and Health-Related Lifestyles in Poland. Health Care Women Int. 22, 207–227. 10.1080/073993301300357160 11814069

[B74] Szklarska A. Jankowska E. A. (2003). Independent Effects of Social Position and Parity on Body Mass Index Among Polish Adult Women. J. Biosoc. Sci. 35, 575–583. 10.1017/S002193200300600X 14621253

[B75] Tobiasz-Adamczyk B. Zawisza K. (2017). Urban-rural Differences in Social Capital in Relation to Self-Rated Health and Subjective Well-Being in Older Residents of Six Regions in Poland. Ann. Agric. Environ. Medenviron. Med. 24, 162–170. 10.26444/aaem/74719 28664687

[B76] Tomescu-Dubrow I. Slomczynski K. M. Sawiński Z. Kiersztyn A. Janicka K. Życzyńska-Ciołek D. (2021). The Polish Panel Survey, POLPAN. Eur. Sociol. Rev. 37, 849–864. 10.1093/esr/jcab017

[B77] Tumin M. M. (1957). Some Unapplauded Consequences of Social Mobility in a Mass Society. Social Forces 36, 32–37. 10.2307/2573743

[B78] Ucieklak-Jeż P. Bem A. (2020). Does "Rural" Always Mean the Same? Macrosocial Determinants of Rural Populations' Health in Poland. Ijerph 17, 397. 10.3390/ijerph17020397 PMC7013667 31936149

[B79] Urbaeva J. (2019). Opportunity, Social Mobility, and Women's Views on Gender Roles in Central Asia. Soc. Work 64, 207–215. 10.1093/sw/swz011 31143954

[B80] van der Waal J. Daenekindt S. de Koster W. (2017). Statistical Challenges in Modelling the Health Consequences of Social Mobility: the Need for diagonal Reference Models. Int. J. Public Health 62, 1029–1037. 10.1007/s00038-017-1018-x 28717828 PMC5668329

[B81] Watkins P. C. Woodward K. Stone T. Kolts R. L. (2003). Gratitude and Happiness: Development of a Measure of Gratitude, and Relationships with Subjective Well-Being. Soc. Behav. Pers. 31, 431–451. 10.2224/sbp.2003.31.5.431

[B82] Watson R. (2004). Research Notes. Państwo i Społeczeństwo 18, 25. 10.7748/ns.18.29.25.s41

[B83] Williams J. C. Blair-Loy M. Berdahl J. L. (2013). Cultural Schemas, Social Class, and the Flexibility Stigma. J. Soc. Issues 69, 209–234. 10.1111/josi.12012

[B84] Williams R. (2006). Generalized Ordered Logit/partial Proportional Odds Models for Ordinal Dependent Variables. Stata J. 6, 58–82. 10.1177/1536867x0600600104

[B85] World Health Organization (2012). Social Inequalities in Health in Poland. World Health Organization Available at: https://www.euro.who.int/en/publications/abstracts/social-inequalities-in-health-in-poland-2012 [Accessed October 14, 2020].

[B86] Zelinska O. Gugushvili A. Bulczak G. Tomescu-Dubrow I. Sawiński Z. Słomczyński K. M. (2021). The Polish Panel Survey (POLPAN) Dataset: Capturing the Impact of Socio-Economic Change on Population Health and Well-Being in Poland, 1988-2018. Data in Brief 35, 106936. 10.1016/j.dib.2021.106936 33786347 PMC7988276

