# Supplementary material for: Social Mobility, Health and Wellbeing in Poland
Source: Front Sociol. 2021 Nov 25;6:736249. doi: 10.3389/fsoc.2021.736249 (PMC8656426; doi:10.3389/fsoc.2021.736249)
Supplement: Supplementary file 1 [file Table1.DOCX]

Supplementary Material

**Table S1: Descriptive statistics**

|  | Mean | SD | Min | Max |
| --- | --- | --- | --- | --- |
| Self-rated health | 3.27 | 1.12 | 1.00 | 5.00 |
| Reported psychological wellbeing | 3.2 | 0.63 | 1.00 | 4.00 |
| Age in 2013 | 49.57 | 19.57 | 21.00 | 91.00 |
| Age^2^ | 2839.78 | 1958.20 | 441.00 | 8281.00 |
| Gender |  |  |  |  |
| *Male* | 0.47 | 0.50 | 0.00 | 1.00 |
| *Female* | 0.53 | 0.50 | 0.00 | 1.00 |
| Marital status |  |  |  |  |
| *Not married* | 0.44 | 0.50 | 0.00 | 1.00 |
| *Married* | 0.56 | 0.50 | 0.00 | 1.00 |
| Urban residence |  |  |  |  |
| *Rural* | 0.41 | 0.49 | 0.00 | 1.00 |
| *Urban* | 0.59 | 0.49 | 0.00 | 1.00 |
| NUTS Region |  |  |  |  |
| *Central* | 0.19 | 0.39 | 0.00 | 1.00 |
| *South* | 0.22 | 0.41 | 0.00 | 1.00 |
| *East* | 0.19 | 0.39 | 0.00 | 1.00 |
| *North-West* | 0.17 | 0.38 | 0.00 | 1.00 |
| *South-West* | 0.08 | 0.28 | 0.00 | 1.00 |
| *North* | 0.15 | 0.36 | 0.00 | 1.00 |
| Height |  |  |  |  |
| *Short* | 0.10 | 0.30 | 0.00 | 1.00 |
| *Tall* | 0.07 | 0.25 | 0.00 | 1.00 |
| Education of respondent |  |  |  |  |
| *Primary* | 0.36 | 0.48 | 0.00 | 1.00 |
| *Secondary* | 0.44 | 0.50 | 0.00 | 1.00 |
| *Tertiary* | 0.21 | 0.41 | 0.00 | 1.00 |

**Table S2.** **Full models for self-rated health and psychological wellbeing, accounting for income**

|  | Model 1:  Self-rated health | Model 2:  Psychological wellbeing |
| --- | --- | --- |
| Intercept | 4.12^***^ (0.30) | 3.97^***^ (0.16) |
| *Intergenerationally immobile* |  |  |
| Professional class | 0.04 (0.14) | -0.05 (0.08) |
| Intermediate class | 0.16 (0.10) | 0.07 (0.05) |
| Working class | -0.20^*^ (0.09) | -0.02 (0.05) |
| *Weight parameters* |  |  |
| Origin | 0.88^*^ (0.38) | 0.98^*^ (0.48) |
| Destination | 0.12 (0.38) | 0.02 (0.48) |
| *Sociodemographic controls* |  |  |
| Age in 2013 | -0.03^**^ (0.01) | -0.02^***^ (0.01) |
| Age^2^ | 0.00^**^ (0.00) | 0.00^**^ (0.00) |
| Gender; 2=female | -0.11 (0.06) | -0.07^*^ (0.03) |
| Married | 0.04 (0.07) | 0.06 (0.04) |
| Urban | 0.16^*^ (0.06) | -0.02 (0.03) |
| *NUTS region* |  |  |
| Central | *Reference* | |
| South | 0.00 (0.09) | -0.04 (0.05) |
| East | -0.04 (0.09) | -0.06 (0.05) |
| North-West | -0.07 (0.10) | -0.13^*^ (0.05) |
| South-West | 0.09 (0.12) | 0.05 (0.06) |
| North | 0.05 (0.10) | -0.03 (0.05) |
| *Mobility dummies* |  |  |
| Downward two-step mobility | -0.07 (0.34) | 0.12 (0.17) |
| Downward one-step mobility | -0.14 (0.15) | 0.09 (0.07) |
| Upward one-step mobility | -0.08 (0.14) | 0.06 (0.05) |
| Upward two-step mobility | 0.04 (0.19) | 0.19^*^ (0.09) |
| *Height* |  |  |
| Short | 0.10 (0.07) | 0.04 (0.03) |
| Tall | 0.26^***^ (0.07) | 0.07 (0.04) |
| *Education* |  |  |
| Primary | -0.17^*^ (0.08) | -0.06 (0.04) |
| Secondary and vocational | *reference* | |
| Tertiary | -0.00 (0.09) | 0.07 (0.05) |
| *Income* |  |  |
| Average household income in 2013 (standardized) | 0.01 (0.03) | 0.00 (0.01) |
| AIC | 4610.23 | 2785.51 |
| BIC | 4737.91 | 2913.69 |
| Number of observations | 1510 | 1542 |

*Notes:* ^*^ *p* < 0.05, ^**^ *p* < 0.01, ^***^ *p* < 0.001, standard errors in parentheses.

|  | Female | Age in 2013 | Education |
| --- | --- | --- | --- |
| Downward two-step mobility | 0.89* | 0.02 | 0.52 |
|  | (0.45) | (0.01) | (0.32) |
| Downward one-step mobility | 0.23 | 0.01 | -0.14 |
|  | (0.19) | (0.00) | (0.14) |
| Upward one-step mobility | -0.01 | 0.00 | -0.08 |
|  | (0.13) | (0.00) | (0.12) |
| Upward two-step mobility | 0.20 | 0.01 | -0.16 |
|  | (0.28) | (0.01) | (0.35) |

**Table S3. Interaction coefficients for self-rated health**

Note: ^*^ *p* < 0.05, ^**^ *p* < 0.01, ^***^ *p* < 0.001. Standard errors in parentheses

**Table S3. Interaction coefficients for reported psychological wellbeing**

|  | Female | Age in 2013 | Education |
| --- | --- | --- | --- |
| Downward two-step mobility | 0.47* | 0.01 | 0.35 |
|  | (0.24) | (0.01) | (0.18) |
| Downward one-step mobility | 0.05 | 0.00 | 0.03 |
|  | (0.1) | (0.00) | (0.08) |
| Upward one-step mobility | 0.02 | -0.00 | 0.01 |
|  | (0.07) | (0.00) | (0.06) |
| Upward two-step mobility | 0.00 | 0.01 | -0.27 |
|  | (0.15) | (0.00) | (0.2) |

Note: ^*^ *p* < 0.05, ^**^ *p* < 0.01, ^***^ *p* < 0.001. Standard errors in parentheses
